# Site-Specific Integration by Circular Donor Improves CRISPR/Cas9-Mediated Homologous Recombination in Human Cell Lines

**DOI:** 10.3390/ijms252011320

**Published:** 2024-10-21

**Authors:** Zhimei Liu, Yue Zhao, Sujun Wu, Shiyu Qi, Yefeng Qiu, Zhengxing Lian

**Affiliations:** 1 Beijing Key Laboratory for Animal Genetic Improvement, National Engineering Laboratory for Animal Breeding, Key Laboratory of Animal Genetics and Breeding of the Ministry of Agriculture, College of Animal Science and Technology, China Agricultural University, Beijing 100193, China; s20193040539@cau.edu.cn (Z.L.); zhaoyuetx@cau.edu.cn (Y.Z.); amywsj@cau.edu.cn (S.W.); bs20223040372@cau.edu.cn (S.Q.); 2 Laboratory Animal Research Center, Academy of Military Medical Science, Beijing 100071, China

**Keywords:** CRISPR/Cas9, NHEJ, rhBCHE, circular donor template, KI efficiency

## Abstract

The technology for obtaining the high-efficiency expression of target proteins through site-specific recombination has made progress. However, using the CRISPR/Cas9 system for site-specific integration of long fragments and the expression of active proteins remains a challenge. This study optimized the linear DNA circularization system, eliminated the prokaryotic plasmid backbone on the traditional foreign gene vector, and generated a homologous arm-free circular donor template with a single guide RNA target site (sgRNA TS). This strategy significantly increased the co-transfection efficiency of the 1.6 kb template and Cas9 plasmid by 1.15-fold, and the average knock-in (KI) efficiency of the 4.7 kb long-fragment template for the two target gene sites increased by 1.3-fold. Subsequently, we used rhBCHE as a reporter gene to efficiently integrate the 5.4 kb fragment containing the gene of interest (GOI) into specific sites in the HEK293T cell line to detect the expression of the circular template at different target sites. Overall, this study further verifies that the length of the circular donor is more conducive to non-homologous integration, and more importantly, we provide a simple and optimized strategy for the construction of long-fragment site integration cell lines.

## 1. Introduction

Currently, overexpressed recombinant protein cell lines are constructed by random integration, transient expression, or site-specific recombination [1]. Randomly integrated cell lines have unclear integration sites and have adverse effects on cell status. Some randomly integrated sites have transcriptional inhibition, resulting in unstable expression levels of recombinant proteins [2]. One of the important problems in the production of recombinant proteins is the yield variability among different clones [3]. Some clones have low expression levels at the beginning of construction, while some clones have high initial expression levels, but the expression levels of most clones will gradually decrease with the extension of culture time [4]. Site-specific recombinant technology to construct stable expression of target protein cell lines has gradually become the main strategy of recombinant protein production [5].

In addition, there are safe harbor loci in the human genome that can be integrated with foreign genes and have no adverse effects on cells, ensuring the viability and proliferation of cells. Moreover, the foreign genes integrated in the safe harbor site can be more stably inherited than those integrated in other target sites (TSs) and are not easily lost [6].

Using CRISPR/Cas9 technology to generate exogenous gene-integrated cell lines is a method to address the above problems. Cas9 nuclease was used to generate specific DNA double-strand breaks (DSBs) in the cell genome, and the foreign gene is integrated into a specific TS of the cell by providing an exogenous gene donor template [7]. In the absence of a homologous repair template, cells are more inclined to choose NHEJ after producing DSBs, which is an efficient repair method with a high repair accuracy [8]. Considering that the homologous repair template containing both sides of homologous arms would reduce the foreign gene load of the donor template, we chose the non-homologous repair template as the delivery template of an exogenous gene. The double-stranded linear DNA donor for non-homologous repair degrades easily after entering cells [9]. In addition, the length of long fragments leads to a low transfection efficiency. Therefore, many studies have proposed solutions to the above problems, e.g., modification at the 5′ end of linear DNA can reduce degradation and improve integration efficiency [9]. Furthermore, the linear DNA template was cyclized to generate a circular DNA double-strand template to improve the transfection efficiency of the long-fragment template [10], which is also a method for improving the donor transfection efficiency.

The donor template structure used in this paper is a circular DNA double-strand template with an sgRNA TS. In addition, we compared the integration efficiency of the circular template and the linear template containing a single sgRNA TS. There was no significant difference, but both were higher than the linear template containing only the target fragment. This result may be attributed to the fact that an sgRNA TS is to some extent guided by Cas9 and enriched in DSB while being cut by Cas9. In conclusion, by optimizing the cyclization system of T4 ligase, we improved the construction efficiency of HEK-293T cell clones with efficient site-specific integration of rhBCHE at different gene loci and stably expressed the active rhBChE.

## 2. Results

### 2.1. Optimization of Self-Circularization System

**Figure 1:**
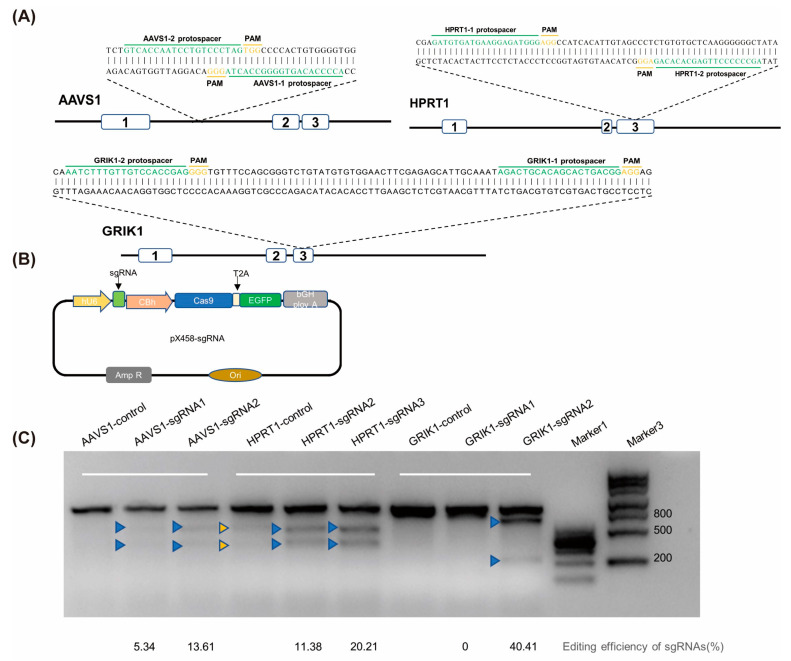
The editing efficiency of different targets. (**A**) Six targets of three gene loci—AAVS1, HPRT1, and GRIK1—were selected. (**B**) The plasmid structure of pX458-sgRNA was constructed. (**C**) T7E1 detection. The plasmid pX458-sgRNA was transfected into cells. The blue arrows represent strips of DNA cut after T7E1 treatment.

We first compared the editing efficiency of sgRNAs in several different genes in HeLa cells. In accordance with the literature and the Benchling website, we selected a total of six sgRNAs targeting AAVS1, HPRT1, and GRIK1 (Figure 1A), and then constructed pX458-sgRNA plasmids and transfected the cells (Figure 1B). The cell genome was extracted 48 h after transfection. Based on T7E1 detection analysis, we were able to judge that the editing efficiency of AAVS1-sgRNA2 and GRIK1-sgRNA2 was relatively higher than other sgRNAs, and we selected two sgRNAs for subsequent experiments (Figure 1C).

**Figure 2:**
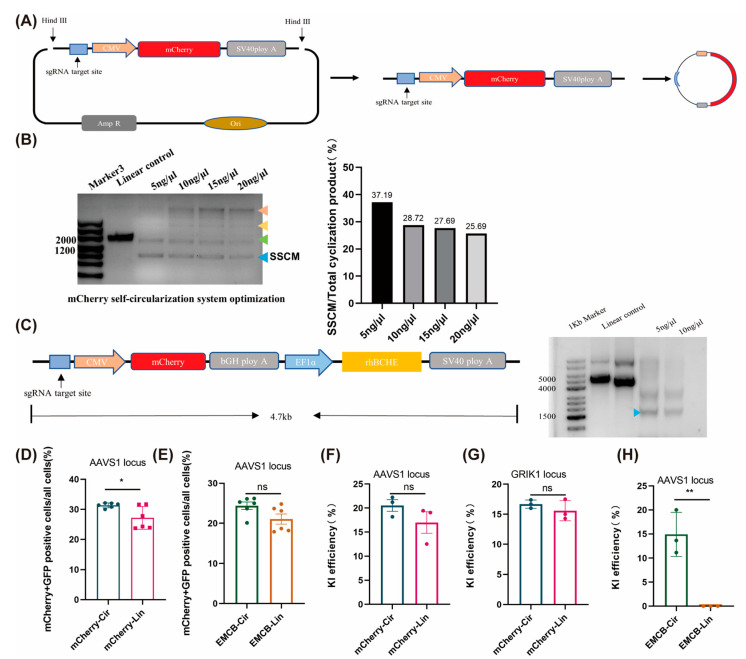
Circular donors improved the percentage of donor-transfected cells. (**A**) mCherry template diagram. The plasmid containing the donor template was cut with Hind III, purified and circularized using T4 ligase. The linear donor template is a PCR product with the same sequence as the donor template. (**B**) Optimization of the circularization system. On the left, different DNA concentration gradients for circularization are shown. The orange arrow indicates the multimolecular Circularization Product 1 (loose), the yellow arrow indicates the multimolecular Circularization Product 2 (helical), the green arrow indicates the nicked single molecule circularization product, and the blue arrow indicates the superhelical single molecule (SSCM). The right image shows the gray-scale value analysis results of SSCM percentage using Image J (version 1.53). (**C**) EMCB template diagram and circularization system. (**D**,**E**) Flow analysis of mCherry and EMCB template transfection efficiency at the AAVS1 locus. The co-transfection efficiency of the circular donor and pX458-sgRNA was significantly higher than that of the linear donor and pX458-sgRNA (N = 6). (**F**) The PCR-verified clone acquisition rate of the mCherry-Cir template at the AAVS1 site of HeLa cells was not significantly higher than that of the mCherry-Lin template, but both were higher than the linear template (mCherry-Lin2) containing only the target fragment (N = 3). (**G**) No significant difference was observed between the mCherry-Cir template and mCherry-Lin template at the GRIK1 site of HeLa cells (N = 3). (**H**) The PCR-verified clone acquisition rate of the EMCB template at the AAVS1 site of HEK-293T (N = 3). (**D**–**H**) Data are presented as the mean ± SD from three independent experiments. Significance was calculated using unpaired Students’ *t*-tests: (ns) *p* > 0.05, (*) *p* < 0.05, (**) *p* < 0.01.

For AAVS1-sgRNA2, for example, we designed a linear donor template containing sgRNA TS (AAVS1-sgRNA2 TS-CMV-mCherry-SV40 pA) (Figure 2A). Different concentration gradients of DNA were designed to further optimize the DNA-cyclized system, and a lower concentration of T4 ligase (2 U/μL) was used. According to the electrophoresis results and ImageJ quantification analysis, the proportion of single-molecule circular DNA in the cyclized product was relatively high at the DNA concentration of 5 ng/μL (Figure 2B). Then, we used the T5 exonuclease to detect the element of circular DNA formation and found that the proportion of monomolecular circular DNA was extremely high and was able to be used for further experiments (Supplemental Figure S1). FACS (BD) results showed that the transfection efficiency of mCherry-Cir was significantly higher (1.15-fold) than that of mCherry-Lin (Figure 2D).

Then, we designed a 4.7 kb donor template (AAVS1-sgRNA2 TS-EF1 α-mCherry-SV40 pA-CMV-rhBCHE-bGh pA) and made it self-circularize at a DNA concentration of 5 ng/μL (Figure 2C). The transfection efficiency of EMCB-Cir was higher than that of EMCB-Lin, but not to a significant level (Figure 2D), which was measured by FACS (BD).

### 2.2. Circular Donor with sgRNA TS Improves the Integration Efficiency of Exogenous Fragment

The circular donor template (GRIK1-sgRNA2 TS-CMV-mCherry-SV40 pA) was constructed based on the optimized cyclization system and the linear template was obtained by PCR. The above two templates were respectively transfected with the pX458-sgRNA plasmid. After 48 h of transfection, FACS was performed and mCherry and EGFP positive cells were sorted to obtain monoclones. After 15 days of monoclonal culture, the cells were visualized using fluorescence microscopy. In total, 60 monoclones in the GRIK1-mCherry-Cir group and 74 monoclones in the GRIK1-mCherry-Lin group DNA with red fluorescence were extracted and verified by 5′/3′ junction PCR. The average integration rates of the GRIK1-mCherry-Cir and GRIK1-mCherry-Lin groups were 16.69% and 15.60%, respectively (Figure 2G).

**Figure 3:**
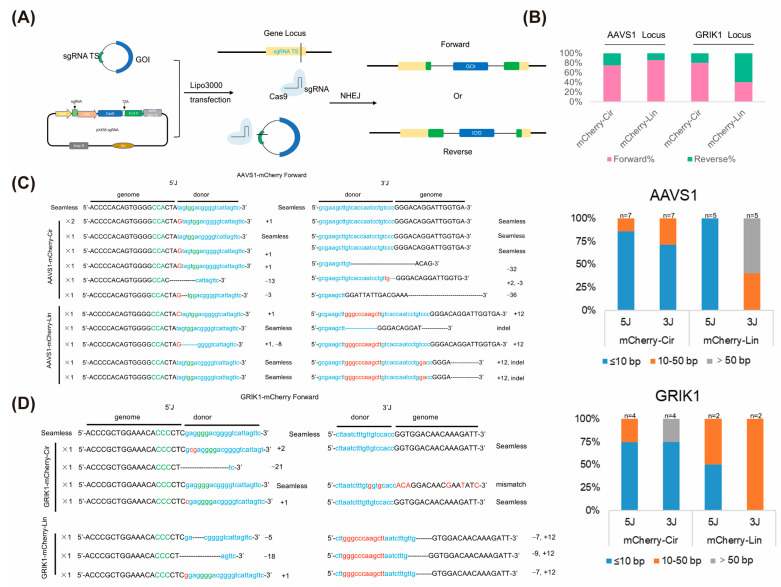
Analysis of integration efficiency and integration patterns of the linear and circular mCherry template. (**A**) Patterns of different integration directions for the circular donor fragment. (**B**) Integrations of the mCherry template at the AAVS1 and GRIK1 loci. (**C**,**D**) Statistics of forward integration indels of the circular and linear mCherry template at different sites.

The template reverse integration tends to form a complete Cas9 TS, which will be cut again by Cas9, leading to a lower proportion of reverse integration compared to forward integration (Figure 3A). Electrophoresis analysis revealed that the forward integration rate of the circular template was 80%, while the reverse integration rate was 20%. In contrast, the forward integration rate of the linear template was 40%, and the reverse integration rate was 60% (Figure 3B). In conclusion, for the GRIK1 gene locus in HeLa cells, the integration efficiency of the 1.6 kb circular template was slightly higher than that of the linear template, and it tended to be forward-integrated.

Subsequently, in order to study whether different gene sites have a bias towards specific template structures, we conducted the above tests for the AAVS1 locus. A total of 99 monoclones in the AAVS1-mCherry-Cir group and 76 monoclones in the AAVS1-mCherry-Lin group and 16 monoclones in the AAVS1-mCherry-Lin2 group with red fluorescence were extracted for 5′/3′ junction PCR validation. The average integration rates for the AAVS1-mCherry-Cir group, AAVS1-mCherry-Lin group, and AAVS1-mCherry-Lin2 group were 20.54%, 17.00%, and 12.5%, respectively (Figure 2F, Supplemental Figure S2). Based on the analysis of the electrophoresis results, the forward integration rate of the circular template was 75%, while the reverse integration rate was 25%. The forward integration rate of the linear template was 85.71%, reverse integration rate was 14.29% (Figure 3B). In conclusion, we found no significant difference in the integration efficiency between the 1.6 kb circular template and linear template at the AAVS1 locus in HeLa cells.

Using the same self-circularization system, we constructed a new 4.7 kb donor template (sgRNA TS-EF1α-mCherry-bGH pA-CMV-rhBCHE-SV40 pA) and used the linear template with the same sequence as a control (Figure 2C). HEK-293T cells were seeded onto a 6-well plate and transfected using Lipofectamine™ 3000. After 48 h of transfection, GFP- and mCherry-positive cells were sorted by flow cytometry. Following 15 days of culture, the surviving cells were lysed and PCR analysis was performed. The PCR results of AAVS1 site indicated that the integration efficiency of the EMCB-Cir circular template was higher than that of the EMCB-Lin template. Therefore, we believe that this circular template was more suitable for the integration of relatively long exogenous fragments (Figure 2F). Furthermore, electrophoresis analysis revealed that the forward-integrated rate was 66.67%, and the reverse-integrated rate was 33.33% (Supplemental Figure S3). In conclusion, the integration efficiency of the 4.7 kb circular template was significantly higher than that of the linear template at the AAVS1 locus in HEK-293T cells.

### 2.3. Circular Donor Results in Different Integration Precision at Junctions

The above target PCR products bands were purified and sequenced to analyze the ligation at the 5′ and 3′ junctions. The results indicated cNHEJ usually results in seamless integration or causes predictable base insertions at the junction. Nevertheless, microhomology-mediated end joining (MMEJ) causes fragment deletion at the junction. In addition, long-fragment indels (other types) were observed in linear and circular template integrated clones. In conclusion, the different integration precision varies at both AAVS1 and GRIK1 sites. As shown in the figure, the 5′ and 3′ junction accuracy produced by mCherry-Cir at the GRIK1 site was higher than that of mCherry-Lin. Furthermore, the 3′ junction accuracy produced by mCherry-Cir at AAVS1 was higher than that of mCherry-Lin, and the 5′ junction accuracy was slightly lower than that of mCherry-Lin.

### 2.4. rhBChE Analysis of Single rhBCHE Copy-Integrated Clones

**Figure 4:**
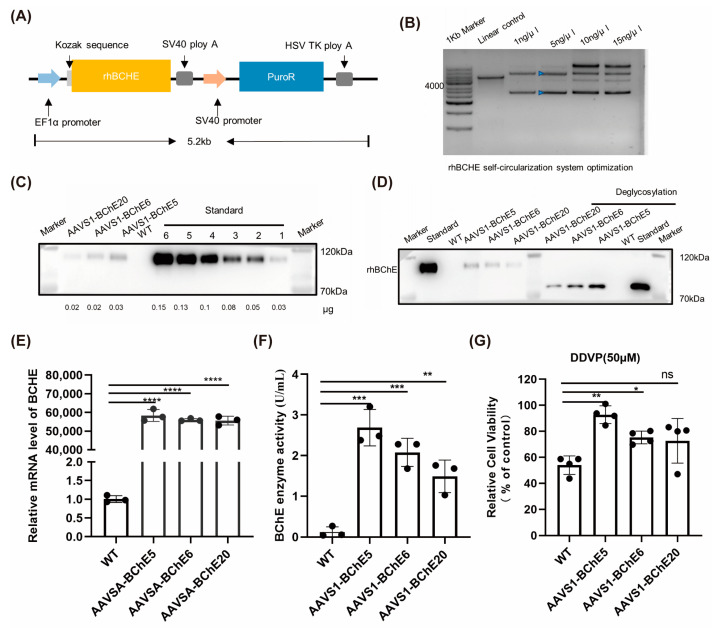
Validation analysis of HeLa cell clones with integrated rhBCHE. (**A**) Schematic diagram of the rhBCHE template. (**B**) Circularization system of the rhBCHE template. Yellow and blue arrows represent multimolecular and monomolecular products, respectively, in the 5 ng/μL circularization system. (**C**) Western blot analysis. After ultrafiltration, the cell supernatant was analyzed by Western blot using different amounts of rhBChE standards as control. (**D**) Deglycosylation assay of the supernatant protein. The molecular weight of the supernatant protein decreased following deglycosylation with rhBChE standard. (**E**) qPCR detection of BCHE transcription levels. (**F**) Analysis of supernatant protein activity. The activity of supernatants protein after ultrafiltration from different cells was determined using the Ellman assay. (**G**) Determination of anti-DDVP activity. A 50 μm concentration was determined as the median lethal concentration dose for WT HeLa cells, and cell viability in other clones was measured relative to this (N = 4). (**C**,**E**,**G**) Data are presented as the mean ± SD from three or four technical replicates. Statistical analysis was performed using an unpaired Student’s *t*-test. (*) *p* < 0.05, (**) *p* < 0.01, (***) *p* < 0.001, (****) *p* < 0.0001.

In subsequent experiments, we used a circular donor template targeting the AAVS1 site in HeLa cells and HEK-293T cells to generate site-specific rhBCHE clones. This approach allowed us to evaluate both the expression level and activity function of the recombinant protein (Figure 4A,B). The average frequency of the obtained desired HeLa cell clones was 24.53%. Cell RNA was extracted for cDNA synthesis and qPCR. The results showed that the expression levels of rhBCHE-integrated clones at the AAVS1 locus were significantly higher compared to those in the WT (Figure 4E).

Thereafter, we spread 1 × 10^5^ cells evenly onto the six-well plate and cultured for 24 h. The DMEM medium containing 10% FBS was replaced with Opti-MEM medium, and the intracellular proteins and supernatant proteins were extracted after an additional 24 h of culture. The supernatant of cell culture was concentrated using a 10 kDa ultrafiltration tube before being prepared for Western blot. The Western blot results for intracellular proteins showed no significant difference between WT and rhBCHE-integrated clones (Supplemental Figure S4A). However, the Western blot results for supernatant proteins showed that while the WT cells had no detectable target size protein band, the site-specific integrated clones had a clear target band (Figure 4C). The reason for this result is that rhBChE is a secreted protein with a signal peptide and undergoes N-glycosylation within the cell before being secreted. However, WT BChE in WT HeLa cells was either not secreted in sufficient quantities or had no necessity to be secreted. Subsequently, we analyzed the glycosylation of rhBChE in the supernatant. After deglycosylation treatment, the specific protein bands were significantly reduced, consistent with the result of the standard product, indicating that rhBChE in the supernatant had correct N-glycosylation (Figure 4D). Based on this result, we also conducted Ellman’s activity assay, which showed that the rhBChE in the supernatant of rhBCHE-integrated clones was significantly higher than in the WT (Figure 4F). In addition, we analyzed the rhBChE activity in the supernatant from five forward-integrated clones and three reverse-integrated clones. Due to the large variability between clones, no significant difference were observed (Supplemental Figure S6). Considering that hBChE is a kind of organophosphorus (OPs) scavenger, we conducted an IC50 test. After DDVP treatment, the cell viability of AAVS1-BChE5 and AAVS1-BChE6 was observably higher than that of WT, indicating that rhBChE had the function of detoxicating OPs when secreted in sufficient amounts (Figure 4G).

### 2.5. rhBChE Analysis of Double rhBCHE Copy-Integrated Clones

**Figure 5:**
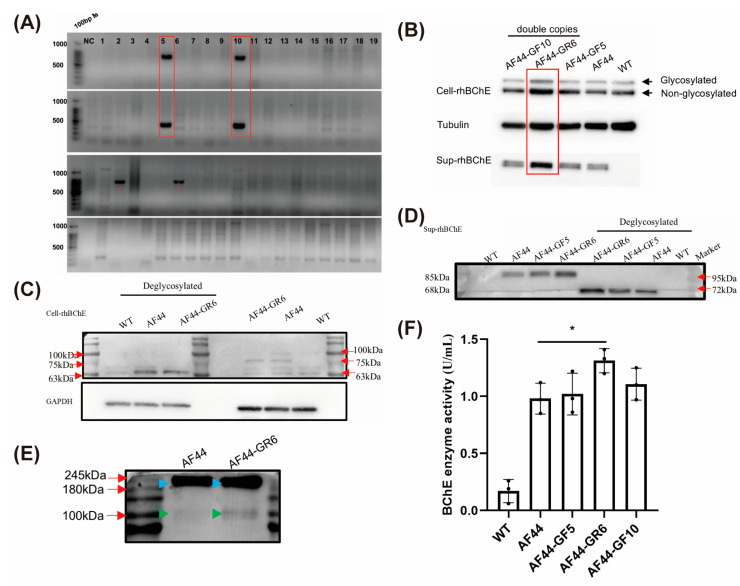
Detection of HEK-293T cell clones with double-copy integration of rhBCHE. (**A**) PCR genotyping of the rhBCHE-forward clones targeting the AAVS1 and GRIK1 loci in HEK-293T cells. The lanes within red rectangles indicate forward knock-in patterns, while the red asterisks indicate reverse knock-in patterns at the GRIK1 locus. (**B**) Western blot analysis. AF44: clone with single-copy forward integration of rhBCHE at the AAVS1 locus; AF44-GF5 and AF44-GF10: clone with single-copy forward integration of rhBCHE at both the AAVS1 and GRIK1 loci; AF44-GR6: clone with single-copy forward integration of rhBCHE at the AAVS1 locus and single-copy reverse integration of rhBCHE at the GRIK1 locus. Tubulin served as the loading control. (**C**) Deglycosylation of the intracellular protein was analyzed by Western blot with GAPDH as the loading control. (**D**) Deglycosylation of the supernatant protein was analyzed by Western blot. (**E**) Multimerization of the supernatant protein was detected by Western blot. The blue arrow indicates the dimer protein band, and the green arrow indicates the monomer protein band. (**F**) Ellman activity assay of the supernatant protein. The rhBChE activity of AF44-GR6 was significantly higher than that of AF44. Data are presented as the mean ± SD from three technical replicates. An unpaired Student’s *t*-test was used for statistical analysis. (*) *p* < 0.05.

We first generated a HEK-293T cell with a single forward-integrated copy of rhBCHE at the AAVS1 site, designated as clone AF44 (Supplemental Figure S4B). Next, we added a single copy of rhBCHE at the GRIK1 site in this clone, resulting in four positive clones. AF44-GF5 and AF44-GF10 were forward-integrated rhBCHE clones. AF44-GR2 and AF44-GR6 were reverse-integrated rhBCHE clones (Figure 5A). We selected AF44-GF5, AF44-GF10, and AF44-GR6 for subsequent experiments. Furthermore, three off-target sites of the AAVS1 and GRIK1 loci were detected, respectively (Supplemental Figure S7). We first assessed rhBChE expression levels in both intracellular and supernatant proteins of the three clones using Western blot. The results showed that the rhBChE bands in the intracellular and supernatant proteins of AF44-GR6 were more prominent than those in the WT and the two forward-integrated clones. In addition, BChE from WT and positive cell clones displayed two distinct bands, representing glycosylated and non-glycosylated protein forms (Figure 5B).

For the above three clones, the supernatant and intracellular proteins were deglycosylated for Western blot analysis. The results demonstrated that the molecular weight of the bands decreased to 68 kDa after deglycation, indicating that both intracellular and supernatant proteins contained glycosylated BChE (Figure 5C,D). AF44 and AF44-GR6 were selected for the analysis of rhBChE polymers. It is well-known that hBChE exists in monomer, dimer, and tetramer forms. The results indicated that the supernatant of AF44 and AF44-GR6 mainly contained dimeric rhBChE and a small amount of monomeric rhBChE (Figure 5E). Subsequently, we measured the activity of rhBChE in the supernatant, and the results revealed that the rhBChE activity in supernatant of AF44-GR6 was significantly higher than that of AF44 (Figure 5F). In conclusion, the expression level and rhBCHE activity of the reverse-integrated clone were significantly higher than those in the single-copy forward-integrated clone.

## 3. Discussion

In this study, we designed an efficient strategy to generate monoclonal cell lines that utilize the NHEJ repair mechanism to integrate target genes into the genome [10]. Compared with previous methods of recombinant protein production, it uses CRISPR/Cas9 technology and circular donor templates to integrate the GOI at the target locus, simplifying the identification of positive monoclonal cells after sorting. The vector is structured as a circular structure of a complete single-copy expression cassette. And it contains an sgRNA TS that could be cut by Cas9, ensuring the vector could be linearized and integrated into the genome [10].

Gene-editing technology has been extensively applied in various fields, including mammalian protein expression [11]. In addition, the traditional integration strategy usually uses NHEJ or homologous directed repair (HDR) to achieve target gene integration [12,13]. But those ways are often limited by low efficiency in generating positive clones, mainly due to poor transfection and integration efficiencies of large DNA fragments. Nevertheless, we discarded the common vector skeleton and retained only the GOI and selection marker gene [14]. After cyclization, the circular helix structure reduced the size of DNA molecules and improved the transfection efficiency. In addition, SSCM does not contain part of the prokaryotes’ elements for entering cells more easily. And SSCM contains the Cas9 cutting site which achieves linearization intracellularly and reduces the probability of degradation of linear fragments.

Our vector structure contains a complete expression cassette for target gene expression, enhancing the flexibility of Cas9 targeting and facilitating the selection of sgRNAs with high editing efficiency and low off-target effects, whereas cNHEJ does not guarantee unidirectional integration [15], but the complete expression cassette can realize both forward and reverse expression of target genes, improving the construction efficiency of positive clones. The expression level of forward and reverse integration clones will be slightly different, and the subsequent studies can further explore how the integration direction of foreign genes influences expression levels.

In this paper, we detailed a simple method for cyclizing linear DNA fragments, verified by T5 exonuclease. In addition, we also found that linear templates with one sgRNA TS exhibit the same integration efficiency as circular templates with one sgRNA TS, and both have a higher integration efficiency than linear templates with only target fragments. We hypothesized that this was due to the short fragment length and traction effect Cas9 exerts on foreign genes containing sgRNA TSs. However, the integration efficiency of the 4.7 kb exogenous circular fragment is significantly higher than that of the linear fragment, due to the overall molecular reduction after cyclization. And further verification of multiple sites and fragments of different lengths is required.

In this study, circular donor integration is mainly mediated by the cNHEJ pathway. Through analyses of junction sequences, we observed various integration patterns in HeLa cells and HEK-293T cells, including cNHEJ- and MMEJ-mediated integration. cNHEJ-mediated integration results in seamless or predictable base insertions at the junction between the donor template and the genome, with the highest proportion at detectable clone junctions [16]. Moreover, since both the Cas9-cleaved genome and donor template probably produce sticky ends, MMEJ-mediated integration using 2 nt microhomologous arms would appear at the junctions [17]. Subsequently, such integration would generate sequence deletion between the microhomologous arms. Nevertheless, the selected target gene locus is the safe harbor locus of the human genome, ensuring the stable integration and expression of foreign genes [6,18,19]. Therefore, partial indels at the junction will not affect the stability of the cell genome and the expression of the target gene.

In conclusion, our study provides an efficient strategy to obtain stable and high-yield recombinant protein-producing cell lines. It serves as a reference for the construction of such cell lines through CRISPR/Cas9-based NHEJ mechanisms and a circular donor template.

## 4. Materials and Methods

### 4.1. Plasmid Construction

AAVS1 sgRNAs, HPRT1, and GRIK1 sgRNAs were selected using the online tool Benchling. The pX458-sgRNA vector was digested with BbsI (NEB, Ipswich, MA, USA). Oligonucleotides corresponding to the sgRNA were annealed and ligated with the linearized pX458-sgRNA vector. All gene-specific sgRNAs were tested using the T7E1 assay and are listed in Supplemental Table S1.

The linear donor mCherry was acquired by PCR amplification (Primestar, Takara, Shiga, Japan) from the pmCherry-N1 plasmid. The sgRNAs’ TSs and HindIII sites were simultaneously added by primer design. The linear donor mCherry was subcloned into pEASY-Blunt (TransGen Biotech, Beijing, China) and digested with HindIII at 37 °C overnight. Enzyme digestion product was subjected to gel electrophoresis and purified to remove the backbone. The purified linear DNA was self-ligated with T4 DNA ligase (NEB, USA) at a concentration of 1.4 ligation units/μL overnight at 16 °C and treated for 10 min at 65 °C to inactivate the ligase. The ligation system was purified to obtain circular donor mCherry for transfection using Lipofectamine™ 3000 (Invitrogen, Carlsbad, CA, USA). Additionally, T5 exonuclease (Beyotime, Haimen, China) was utilized to eliminate to remove the linear fragment and nicked circular template from the template mixture for further purification. The reaction was set up by adding nuclease-free water (to a total volume of 10 μL), 1 μL of 10X reaction buffer, 500 ng of DNA, and 0.5 μL of T5 exonuclease to a 200 μL centrifuge tube. After incubation at 37 °C for 30 min, the mixture was placed on ice. To terminate the reaction, 0.2 μL of 0.5 M EDTA (Beyotime, China) was added, and then 2 μL of 6X DNA Loading Buffer was added for 2% agarose gel electrophoresis. The system can be scaled up in proportion.

The linear BCHE fragment was obtained by PCR amplification from the pPB-BCHE-Puro^r^ plasmid. The same aforementioned method was used to generate the circular rhBCHE donor. All primers are listed in Supplemental Table S2.

### 4.2. Cell Culture and Transfection

HeLa cells and HEK-293T cells were cultured in DMEM/High Glucose and DMEM (Gibco, Norristown, PA, USA), respectively, supplemented with 10% fetal bovine serum (FBS) and incubated at 37 °C in a 5% CO_2_ atmosphere. In total, 5 μg of sgRNAs-specific pX458-sgRNA with 2.5 μg of either circular or linear donor was cotransfected into about 10^6^ cells separately using Lipofectamine™ 3000. Two days post-transfection, transfected cells were sorted by fluorescence-activated cell sorting (FACS). GFP-positive single cells were collected for rhBCHE-integrated clones and GFP/mCherry double-positive single cells were collected for mCherry-integrated and EMCB-integrated clones, respectively.

### 4.3. Fluorescence Observation and PCR Verification

After the transfection of sgRNAs-specific pX458-sgRNA for 48 h, we fixed the cell via 4% paraformaldehyde (Solarbio, Beijing, China) in a 48-well plate and stained with DAPI (Solarbio, China). The fixed cells were then observed via a fluorescence-inverted microscope to assess the efficiency of sgRNAs-specific pX458-sgRNA.

Two weeks post-FACS, single clones were observed via a fluorescence-inverted microscope for counting the efficiency of mCherry-positive clones. All surviving clones were lysed using a lysis solution for PCR amplification to detect the genome–donor junction. PCR products were visualized by gel electrophoresis and products of positive clones were directly purified and sequenced. The primers for 5′ and 3′ junction PCR are listed in Supplemental Table S3.

### 4.4. qRT-PCR

Cell RNA was extracted using the RN28-EASYspin Plus kit (Aidlab, Beijing, China) and reverse-transcribed into cDNA using PrimeScript™ RT Reagent Kit with gDNA Eraser (Takara, Japan). qRT-PCR was performed using PC60-2×SYBR Green qPCR Mix Low ROX (Aidlab, China) on a real-time PCR system. The primers used for qRT-PCR are listed in Supplemental Table S4.

### 4.5. Western Blot Analyses

Cultured rhBCHE-positive clones were harvested and resuspended in RIPA lysis buffer (Beyotime, China). Cell culture supernatant was collected after incubation for 48 h to determine BChE activity and perform Western blot, separately. The protein concentration of each extract was quantified using the BCA Protein Assay Kit (Beyotime, China) according to the manufacturer’s protocols. The crude lysate was mixed with SDS buffer and incubated at 95 °C for 10 min. Equal amounts of protein samples (approximately 20–30 μg each) were loaded into separate lanes of 8% gel for SDS–polyacrylamide gel electrophoresis (Bio-Rad, Hercules, CA, USA). Recombinant human BChE (Sinobiological, Beijing, China) was used as a control. After electrophoretic separation, proteins were transferred to a polyvinylidene fluoride membrane (Millipore, Bedford, MA, USA). The membranes were blocked with 5% milk powder for 1 h at room temperature. A 1:1000 dilution of rabbit monoclonal antibody to BChE (Abcam, Cambridge, UK) was used as the primary antibody and incubated at 4 °C overnight. The membranes were washed thoroughly with Tris-buffered saline containing 0.1% Tween 20 (TBST). A 1:10,000 dilution of goat anti-rabbit IgG-HRP (Beyotime, China) was used as the secondary antibody and the membrane was incubated at 4 °C for 1 h. The quantification of protein purity was performed by ImageJ (US National Institutes of Health, Bethesda, MD, USA).

### 4.6. Deglycosylation

The cell culture supernatant of each group was collected after incubation for 24 h. Deglycosylation of BChE was then performed using PNGase F (NEB, USA) according to the manufacturer’s protocols.

### 4.7. BChE Activity Assay

The cell culture supernatant of each group was collected after incubation for 48 h. BChE activity was determined using a modified Ellman’s method. Briefly, a standard from the BChE activity assay kit (Nanjing Jiancheng, Nanjing, China) was used to generate a standard curve. Next, a 30 μL sample of each clone from three replicate wells was added to 96-well plate to test BChE activity according to the manufacturer’s protocols.

### 4.8. IC50

Cells of each clone and WT HeLa were plated into a 96-well plate, and DDVP (O,O-dimethyl-2,2-dichlorovinyl phosphate) (Agro-Environmental Protection Institute, Ministry of Agriculture and Rural Affairs, Beijing, China) was added. After 48 h, cell activity was assessed using an MTT kit (Solarbio, Beijing, China) and the IC50 (half maximal inhibitory concentration) curve was drawn. Additionally, the cell activity of each positive clone was determined at the IC50 of WT HeLa cells.

## 5. Conclusions

In this study, we optimized a system for circularizing double-stranded DNA and determined that the single-molecule supercoiled product accounted for the highest proportion at a concentration of 5 ng/μL. We also proposed the use of T5 exonuclease to digest excess products to increase the proportion of SSCM. SSCM containing specific Cas9 cleavage sites can be linearized and integrated into the same genomic target in the presence of Cas9, theoretically enhancing specificity and reducing off-targets. However, the off-target situation may vary depending on the target, and further sequencing verification is required. Our research has advanced a method for the efficient preparation of cell lines with integrated target genes.

## Data Availability

All data supporting the findings of this study are available in the article or in the supplementary data files, or are available from the corresponding author upon request. All raw data used in the study have been deposited at Figshare and are available at https://doi.org/10.6084/m9.figshare.22566607 (accessed on 1 January 2024).

## Supplementary Materials

The following supporting information can be downloaded at: https://www.mdpi.com/article/10.3390/ijms252011320/s1.

## Abbreviations

CRISPR	clustered regularly interspaced short palindromic repeats
Cas9	CRISPR-associated protein 9
sgRNA	single guide RNA
sgRNA TS	sgRNA target site
DSBs	DNA double-strand breaks
NHEJ	non-homologous end joining
GOI	gene of interest
rhBChE	recombinant human butyrylcholinesterase
OPs	organophosphorus
OPNAs	organophosphorus nerve agents
HDR	homologous directed repair
MMEJ	microhomology-mediated end joining
SSCM	superhelical single molecule
