# Supplementary material for: Site-Specific Integration by Circular Donor Improves CRISPR/Cas9-Mediated Homologous Recombination in Human Cell Lines
Source: Int J Mol Sci. 2024 Oct 21;25(20):11320. doi: 10.3390/ijms252011320 (PMC11508559; doi:10.3390/ijms252011320)
Supplement: Supplementary file 1 [file ijms-25-11320-s001.zip › ijms-3200021-supplementary.pdf]

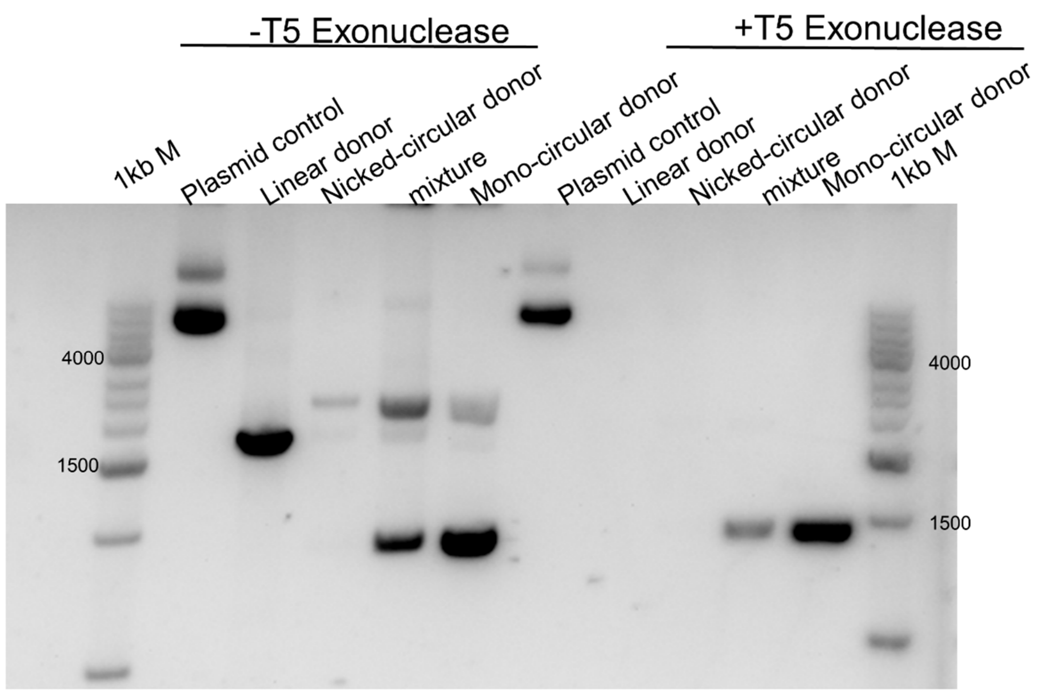


**Supplemental Fig S1.** T5 exonuclease was used to detect the element of circular DNA formation.

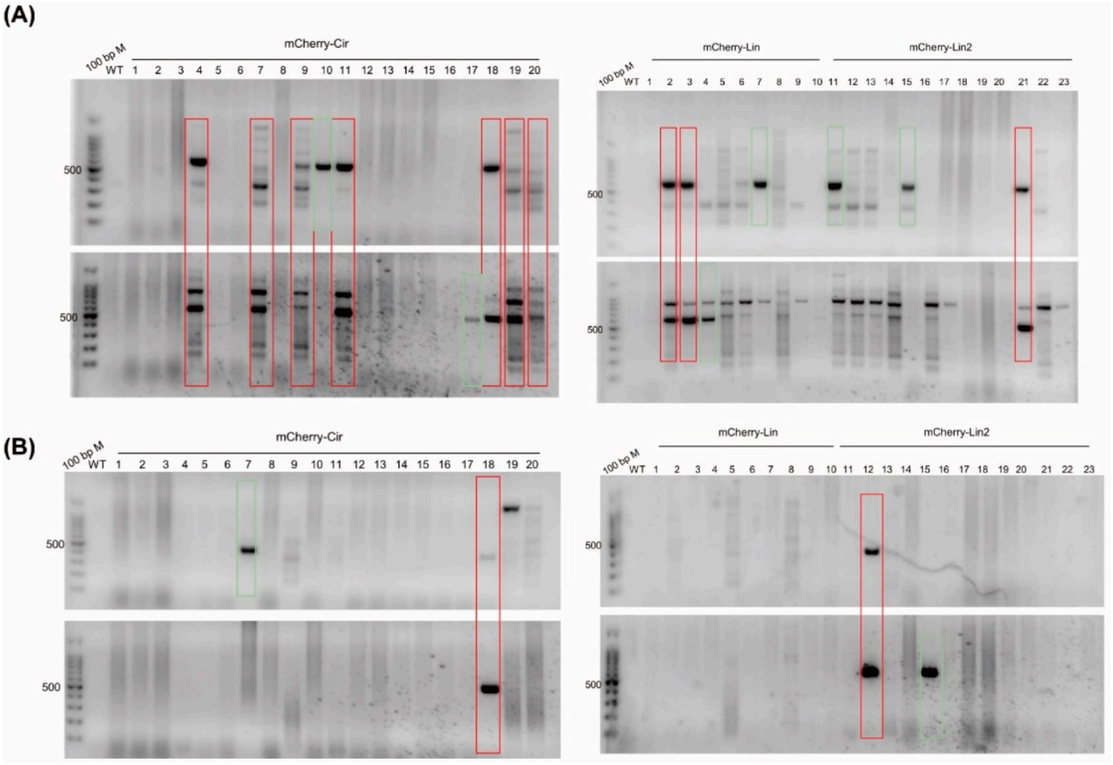


**Supplemental Fig S2.** mCherry donor template integration was assessed through 5’/3’junctions PCR. (A) and (B) Forward and reverse PCR genotyping results of the fluorescence-positive clones for mCherry-Cir, mCherry-Lin and mCherry-Lin2 donors. The lanes with red rectangles indicated mCherry-integrated clones, and the green rectangles indicated one-sided integration. Integration rates for mCherry-Cir and mCherry-Lin were higher than those for mCherry-Lin2 which lacks the

target site.

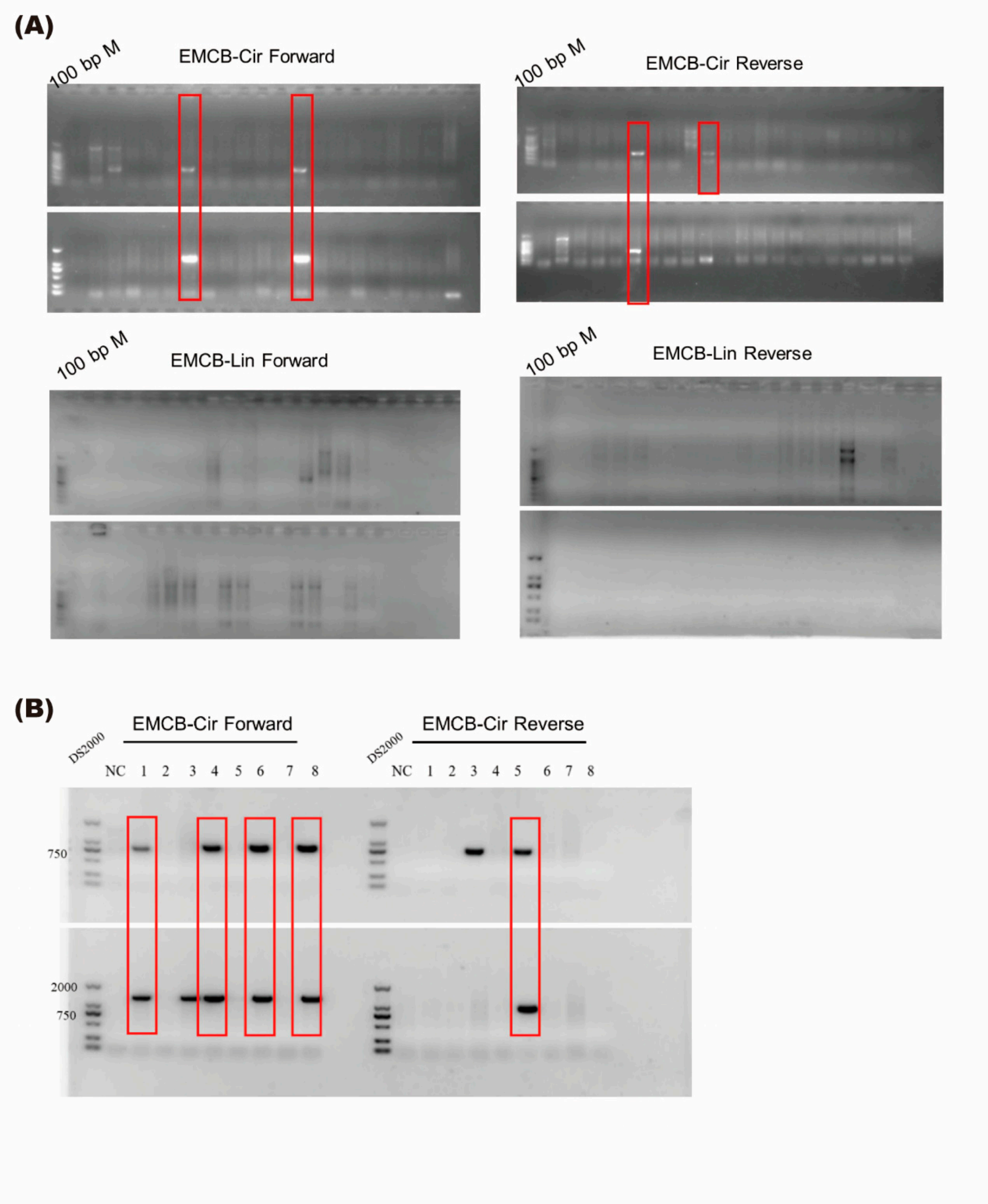


**Supplemental Fig S3.** EMCB donor template integration was assessed through 5'/3'junctions PCR. (A) PCR genotyping results of the fluorescence-positive clones using EMCB donors in first experiment. The top two figures showed the forward and reverse PCR assay results of EMCB-Cir, revealing three positive clones at both 5' and 3' junctions and one positive clone at the 5' junction. The subsequent two figures showed the forward and reverse PCR assay results of EMCB-Lin, which showed no positive clones. The lanes with red rectangles indicated clones with EMCB circular donor integration. And there was no fluorescence-positive clones were found in EMCB linear donor integration group. (B) PCR genotyping results of the fluorescence-positive clones using EMCB donors in second experiment. No red fluorescent clones were found in the EMCB-Lin group, while the EMCB-Cir assay results showed there were four forward-integrated clones and one reverse-

integrated clone. There were also two unilaterally positive clones.  The integration efficiency of the EMCB-Cir group was higher than that of EMCB-Lin group.

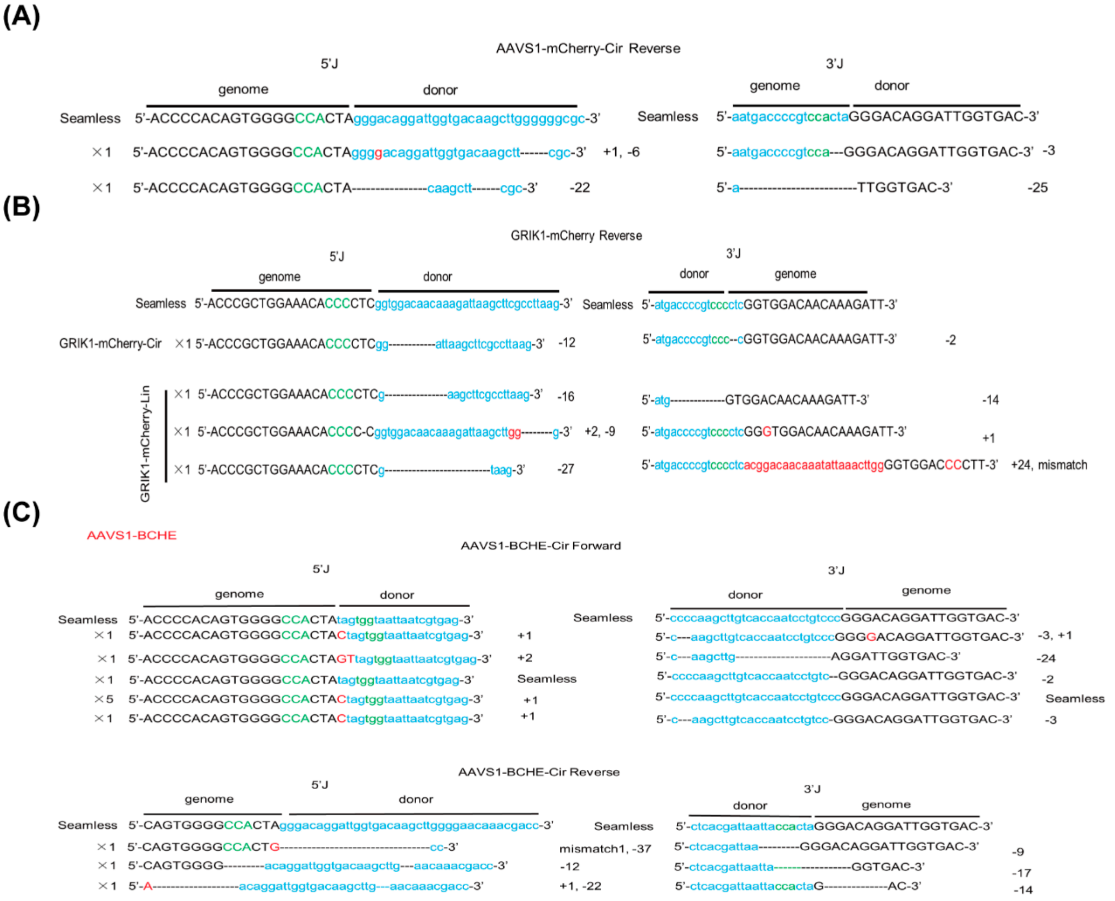


**Supplemental Fig S4.** 5'/3'junctionsintegration sequence analysis of partial clones**.** (A) and (B) Statistics on the reverse integration indels of circular and linear mCherry templates at different sites. (C) Statistics of forward and reverse integration indels for circular rhBCHE template at AAVS1 site. The term "seamless" referred to  the sequence where the vector and genome were seamlessly connected and acted as a reference. Genome sequence was marked in black.The vector sequence was identified in blue. PAM sequence was green. Dashed lines indicated sequence deletions and red sequences indicated base insertions or mismatches.

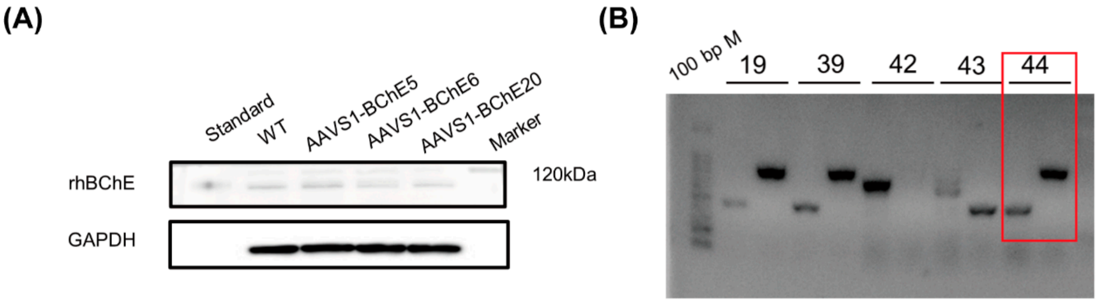


**Supplemental Fig S5.** Detection of the rhBCHE-positive clones. (A)Western blot results of intracellular proteins. (B) PCR genotyping results indicated the rhBCHE-positive HEK-293T clones using rhBCHE-Cir donors at AAVS1 locus.

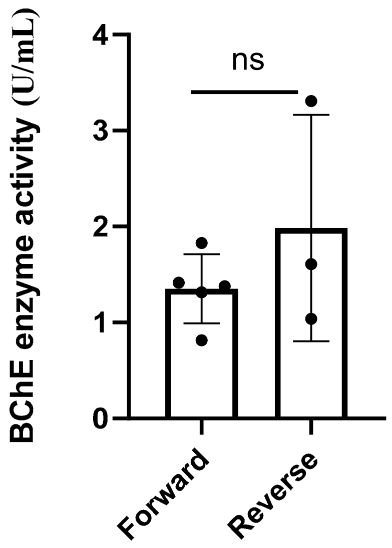


**Supplemental Fig S6.** rhBChE activity analysis of 5 forward-integrated clones and 3 reverse-integrated clones. Although no significant difference were observed in the expression levels of recombinant proteins between forward-integrated clones and reverse-integrated clones due to inter-clonal variability, but it is noteworthy that the expression level of reverse-integrated clones was higher than that of forward-integrated clones. Forward group: N=5. Reverse group: N = 3 biological replicates. Error bars, SD from three or five biological replicates. Significance was calculated using unpaired Student's t-tests: (ns) $p > 0.05$.

**(A)**

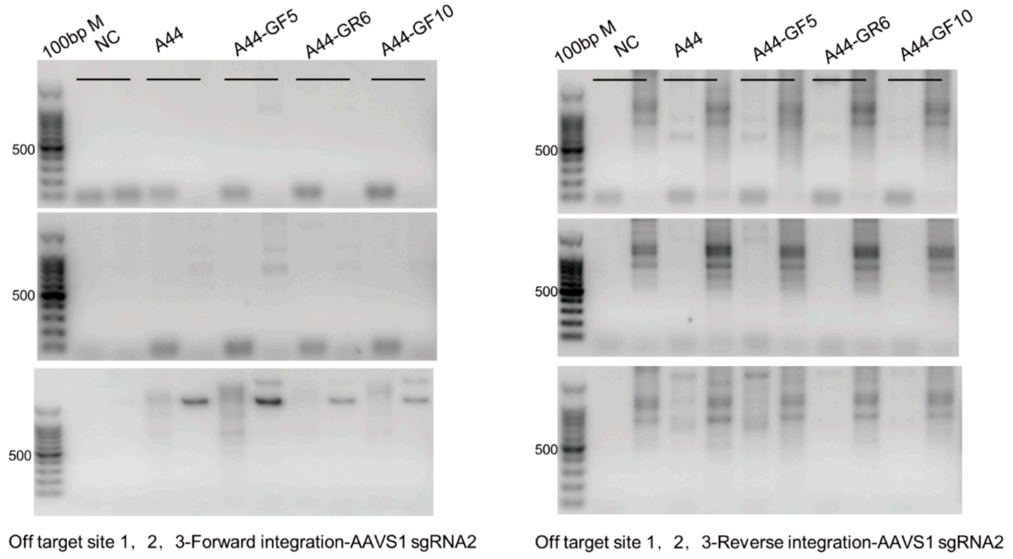


**(B)**

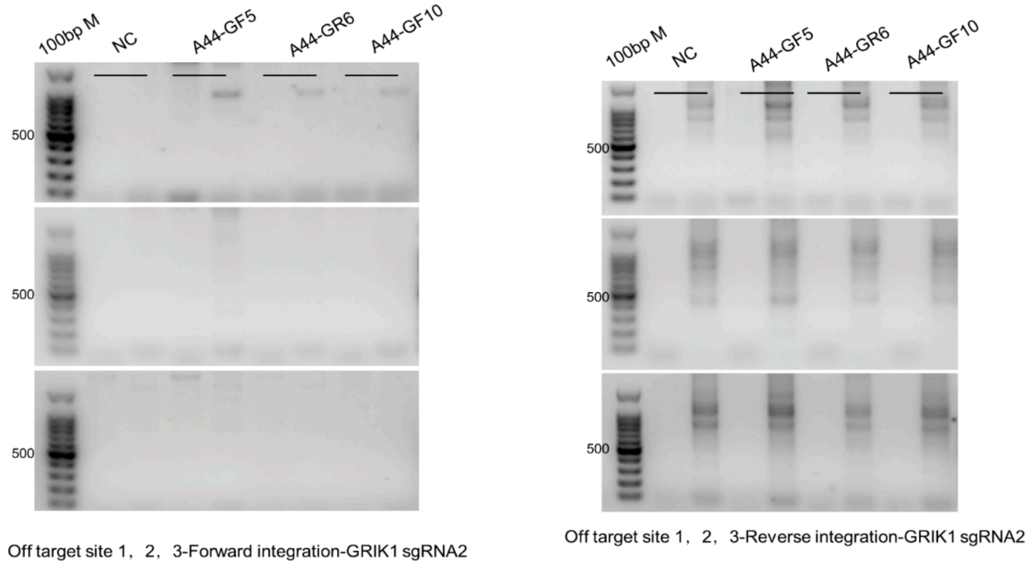


**Supplemental Fig S7.** (A) and (B) Off-targets detection for AAVS1 and GRIK1 loci. The Cas-Offinder website was used to predict three possible off-target sites for AAVS1 sgRNA2 and GRIK1 sgRNA2, and PCR was used to detect the off-target status of each clone. As shown in the figure, no off-target was detected in the positive clones.

**Supplemental Table S1. Target genes and oligos selected for T7E1 detection in HeLa cells.**

| Primer name | Sequence | PAM |
|---|---|---|
| AAVS1-F-1 | CACCGACCCCACAGTGGGGCCACTA | GGG |
| AAVS1-R-1 | AAACTAGTGGCCCCACTGTGGGGTC | |
| AAVS1-F-2 | CACCGTCACCAATCCTGTCCCTAG | TGG |
| AAVS1-R-2 | AAACCTAGGGACAGGATTGGTGAC | |
| HPRT1-F-1 | CACCGATGTGATGAAGGAGATGGG | AGG |
| HPRT1-R-1 | AAACCCCATCTCCTTCATCACATC | |
| HPRT1-F-2 | CACCGAGCCCCCCTTGAGCACACAG | AGG |
| HPRT1-R-2 | AAACCTGTGTGCTCAAGGGGGGCTC | |
| GRIK1-F-1 | CACCGAGACTGCACAGCACTGACGG | AGG |
| GRIK1-R-1 | AAACCCGTCAGTGCTGTGCAGTCTC | |
| GRIK1-F-2 | CACCGAATCTTTGTTGTCCACCGAG | GGG |
| GRIK1-R-2 | AAACCTCGGTGGACAACAAAGATTC | |

**Supplemental Table S2. Primers used for plasmid construction. All oligos are in the 5' to 3' direction.**

| Primer name | Sequence |
|---|---|
| BCHE-AF | CCCAAGCTTGTCACCAATCCTGTCCCTAGTGGtaATTAAtcgtgaggctccggtgcc |
| BCHE-GF | CCCAAGCTTAATCTTTGTTGTCCACCGAGGGGtaATTAAtcgtgaggctccggtgcc |
| BCHE-R | CCCAAGCTTGAACAAACGACCCAACACCGTG |
| mCherry-GF | CCCAAGCTTAATCTTTGTTGTCCACCGAGGGGacggggtcattagttcatagcc |
| mCherry-AF | CCCAAGCTTGTCACCAATCCTGTCCCTAGTGGacggggtcattagttcatagcc |
| mCherry-R | CCCAAGCTTCGCCTTAAGATACATTGATGAG |
| EMCB-AF | CCCAAGCTTGTCACCAATCCTGTCCCTAGTGGggctccggtgcccgtcagtg |
| EMCB-AR | CCCAAGCTTAGAGAGGGAGTGGCCAACTC |

**Supplemental Table S3. Primers used for integration detection.**

| Primer name | Sequence | Gene of interest | Target site |
|---|---|---|---|
| 5FB-A2-F2 | CCCTGTCATGGCATCTTC | rhBCHE | AAVS1 |
| 5FB-A2-R2 | ATCACTTTCCCAGTTTACCC | | |
| 3FB-A2-F2 | GGTGCCTGAGATAAACGC | | |
| 3FB-A2-R2 | GAAAGGTGAAGAGCCAAAGT | | |
| 5RB-A2-F | CTTTCTTTGCCTGGACACCC | | |
| 5RB-A2-R2 | CCTGAGATAAACGCGGGACT | | |
| 3RB-A2-F | CCATAACCCGTAAAGAGGC | | |
| 3RB-A2-R2 | GAGACTAGGAAGGAGGAGGC | | |

| Primer name | Sequence | Gene of interest | Target site |
|---|---|---|---|
| 5FB-G2-F | CCTTGTCCTTGGGCATCA | rhBCHE | GRIK1 |

| | |
|---|---|
| 5FB-G2-R | TCGCAGCAGGTCATCAAA |
| 3FB-G2-F | CGAAATGACCGACCAAGC |
| 3FB-G2-R | GTCCCATACCTGTGCTGTC |
| 5RB-G2-F | GACCAACCGTATTTCCTCAA |
| 5RB-G2-R | CGGTGCCTGAGATAAACG |
| 3RB-G2-F | GAAAGCAGCGAGACAGGC |
| 3RB-G2-R | GCAGACATGGTCGGGTGA |

| Primer name | Sequence | Gene of interest | Target site |
|---|---|---|---|
| 5F-BM-F1 | CCCTGTCATGGCATCTTC | | |
| 5F-BM-R1 | ATCACTTTCCCAGTTTACCC | | |
| 3F-BM-F1 | CCCACTGTCCTTTCCTAA | | |
| 3F-BM-R1 | AACTCCCTGCTGGTCTCA | rhBCHE | AAVS1 |
| 5R-BM-F1 | TCTTCTTCCTCCAACCCG | | |
| 5R-BM-R1 | TTGCCAGCCATCTGTTGT | | |
| 3R-BM-F1 | CCATAACCCGTAAAGAGGC | | |
| 3R-BM-R1 | GAGACTAGGAAGGAGGAGGC | | |
| 5F-BM-F2 | GTTCTCCTGTGGATTCGGGTCA | | |
| 5F-BM-R2 | CGAAAGCAGCGAGACAGGCGCGAAG | | |
| 3F-BM-F2 | CTTGGGTGATGTTGTTGG | | |
| 3F-BM-R2 | GAAAGGTGAAGAGCCAAAGT | | |
| 5R-BM-F2 | GTTCTCCTGTGGATTCGGGTCA | | |
| 5R-BM-R2 | GGAGTGGAAAGCAGGATTCCATC | | |
| 3R-BM-F2 | GAAAGCAGCGAGACAGGCGCGAAGG | | |
| 3R-BM-R2 | GAAAGGTGAAGAGCCAAAGT | | |

| Primer name | Sequence | Gene of interest | Target site |
|---|---|---|---|
| 5F-A-F | TCTTCTTCCTCCAACCCG | | |
| 5F-A-R | TGAGTCAAACCGCTATCC | | |
| 3F-A-F | AGATCATAATCAGCCATACCA | | |
| 3F-A-R | AGTGAAACGCACCAGACA | | |
| 5R-A-F | TCTTCTTCCTCCAACCCG | | AAVS1 |
| 5R-A-R | GATCATAATCAGCCATACCAC | | |
| 3R-A-F | TGAGTCAAACCGCTATCC | | |
| 3R-A-R | TGGCTCCATCGTAAGCAAACCT | mCherry | |
| 5F-G-F | CCAACCGTATTTCCTCAA | | |
| 5F-G-R | CCAAGTGGGCAGTTTACC | | |
| 3F-G-F | CATAATCAGCCATACCAC | | |
| 3F-G-R | TAAAAGGATCGTTAGAAAG | | GRIK1 |
| 5R-G-F | CCAACCGTATTTCCTCAA | | |
| 5R-G-R | TCATAATCAGCCATACCAC | | |
| 3R-G-F | TAATAGCGATGACTAATACG | | |
| 3R-G-R | GTGAGTCCCATACCTGTG | | |

**Supplemental Table S4. Primers used for qRT-PCR.**

| Primer name | Sequence |
|---|---|
| β-Actin-F | AGATGTGGATCAGCAAGCAG |
| β-Actin-R | CCAATCTCATCTCGTTTTCTG |
| rhBChE-F | TTTAATGCTCCTTGGGCGGT |
| rhBChE-R | TTTACTGACAAAGGAGTCCCAT |

**Supplemental Table S5. Off-targets detection of AAVS1 and GRIK1 locus.**

| Sites name | Sequence |
|---|---|
| AAVS1 sgRNA2 Off Target Site1 | GCCACAAATCCTGTCCCTGGAGG |
| AAVS1 sgRNA2 Off Target Site2 | GCCACCACTCCTGTCCCTGGTGG |
| AAVS1 sgRNA2 Off Target Site3 | GCCACTAATACTGTCCCTAGCGA |
| GRIK1 sgRNA2 Off Target Site1 | AATCTTTGGTGTCCACAGACAGG |
| GRIK1 sgRNA2 Off Target Site2 | AACCTTTGTTTTCCACCCAGTGG |
| GRIK1 sgRNA2 Off Target Site3 | AAGCTTTGTTGCCCACCGAGCGA |
